# Supplementary material for: Stimulus duration encoding occurs early in the moth olfactory pathway
Source: Commun Biol. 2024 Oct 3;7:1252. doi: 10.1038/s42003-024-06921-z (PMC11449909; doi:10.1038/s42003-024-06921-z)
Supplement: Supplementary file 2 — Supplementary Figs. [file 42003_2024_6921_MOESM2_ESM.pdf]

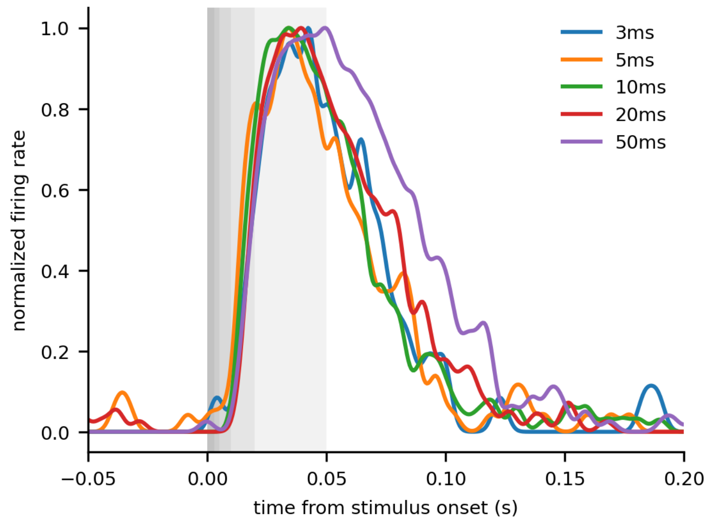

Supplementary Figure 1. **Pulse response.** The normalized average firing rate response is almost identical for all stimuli in the range 1 ms to 50 ms. Shaded areas indicate the stimulus duration, darkest shading indicating the shortest stimulus (3 ms). The firing rate profiles were obtained by kernel density estimation with $\sigma = 3\,\mathrm{ms}$.

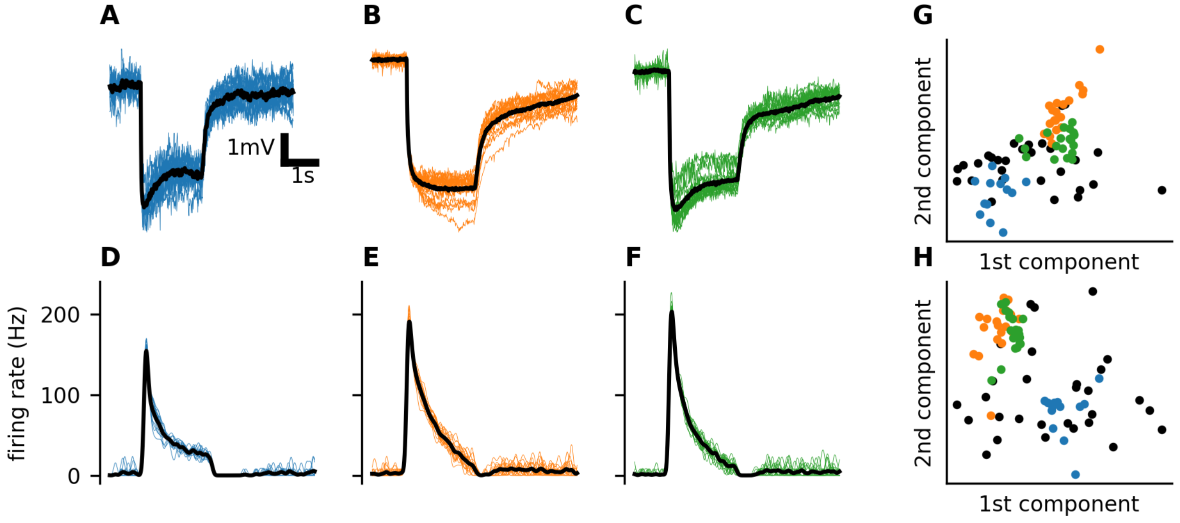

Supplementary Figure 2. **Heterogeneity of ORN responses. A-C:** LFP in response to a 2 s stimulus. Each panel (color) corresponds to a single neuron presented several times with the same stimulus. Thin lines are the individual trials, the black lines represent their average. **D-F:** Firing profiles of the three different neurons. Colors represent the neuron, as in **A-C**. Thin lines are the individual trials, the thick line represents their average. **G:** Scatter plot of the first two PCA components of the LFP. Each black point corresponds to a different neuron, while each of the colored points represents a single trial of one of the three neurons from **A-C**. The colored points are always concentrating around one spot, indicating that the responses of each neuron are stable in time and do not capture the heterogeneity of the whole population. **H:** Same as **G**, but for the firing rate profiles.

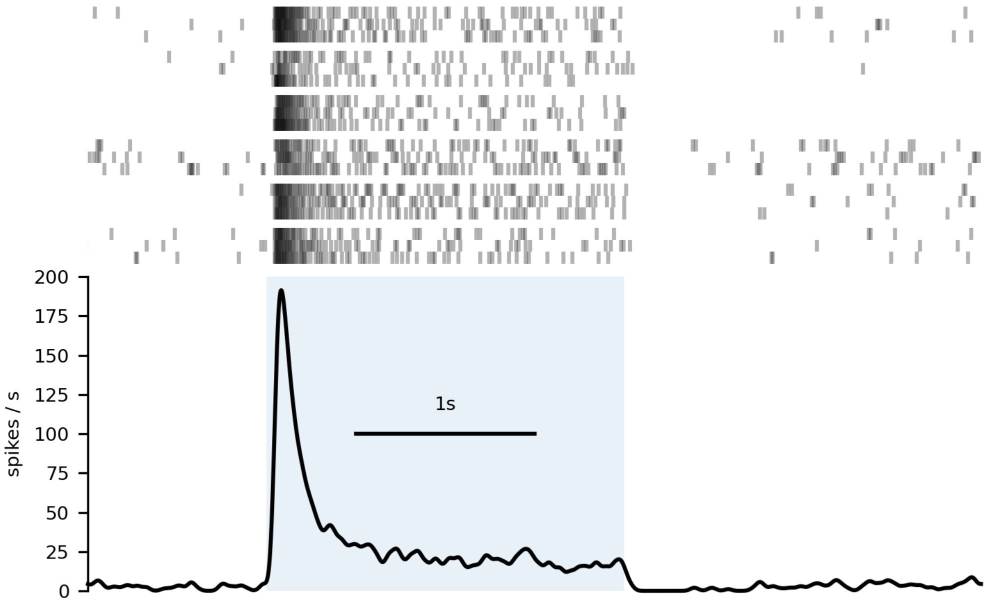

Supplementary Figure 3. **Constant mechanical pressure control.** We added a second electrovalve to deliver non-odorized air. This valve was in the opposing phase to the valve that delivers odor stimuli so that the airflow sent to the antenna was constant before, during, and after stimuli. We observed the same inhibitory phase after the stimulus offset, indicating that the inhibitory phase was not a mechanical artifact.

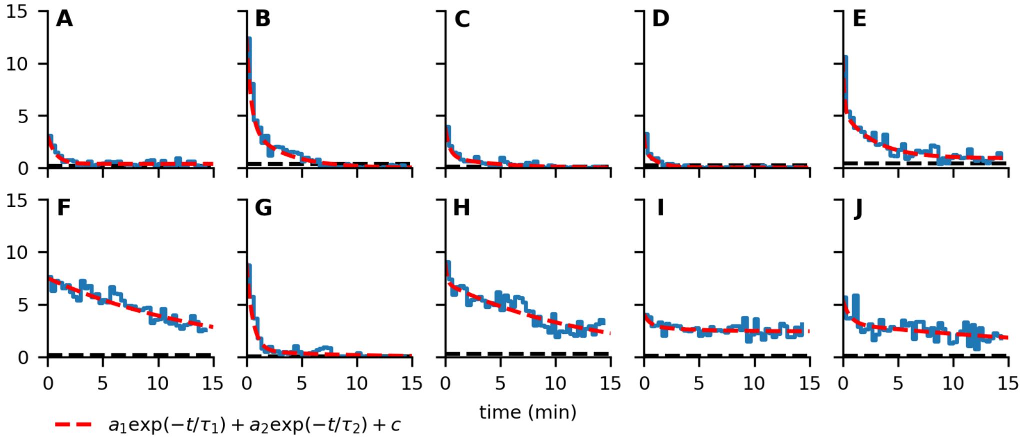

dose 10 pg

	a_1	τ_1	a_2	τ_2	c	spont.
A	2.69	43.66	0.00	43.3	0.39	0.18
B	8.66	25.96	3.83	202.0	0.00	0.36
C	2.76	23.24	1.12	266.6	0.00	0.13
D	1.51	5.52	1.70	67.5	0.04	0.27
E	5.16	8.53	4.52	169.1	0.93	0.44

dose 1 ng

	a_1	τ_1	a_2	τ_2	c	spont.
F	0.09	0.41	7.51	927.1	0.00	0.16
G	8.36	34.58	0.63	462.4	0.00	0.03
H	2.02	10.46	7.04	789.6	0.00	0.31
I	1.12	32.05	0.52	233.8	2.43	0.09
J	2.27	39.85	3.14	1672.8	0.00	0.09

Supplementary Figure 4. **Sustained firing activity measured over long periods.** We first measured the spontaneous activity during a 15 min period (black dashed lines) and then stimulated the ORN with either 10 pg (**A-E**) or 1 ng (**F-J**) of pheromone. Blue lines indicate the firing rate as measured by counting spikes in 20 s bins, starting at 3 s after the pulse offset. Red dashed lines show a double exponential fit. The ORNs stimulated with a 1 ng dose did not return to or near their spontaneous activity within the 15 min period (except for **G**). The table shows the fitted parameters. a_1, a_2, c and the spontaneous activity are given in Hz, b_1 and b_2 are in seconds.

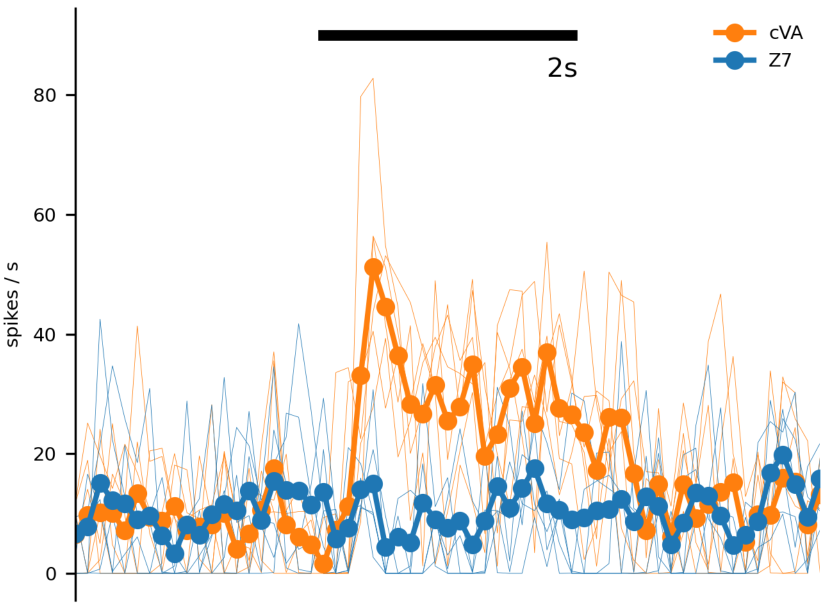

Supplementary Figure 5. **T1 neurons in *Drosophila* do not respond to Z7-12:Ac.** Stimuli lasted 2 s, with 100 ng of Z7-12:Ac and 10 µg of cVA, $n = 6$ ORNs.

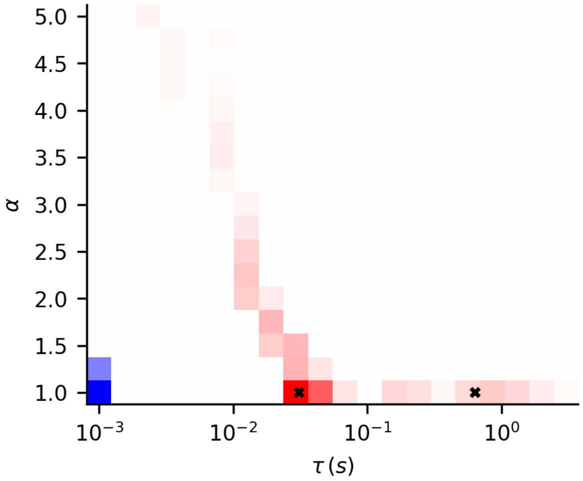

Supplementary Figure 6. **Lasso regression coefficients.** Color-coded are the coefficients obtained from the lasso regression. Red indicates positive values, blue indicates negative values. Note that LFP is negative, and therefore negative coefficients indicate positive firing response. Non-zero values concentrate around three locations, close to $\alpha = 1$. We narrowed down the optimization to two adaptation time constants and found that the adaptation is well represented by time constants 31 ms and 635 ms (indicated by black crosses).
