# Supplementary material for: Stimulus duration encoding occurs early in the moth olfactory pathway
Source: Commun Biol. 2024 Oct 3;7:1252. doi: 10.1038/s42003-024-06921-z (PMC11449909; doi:10.1038/s42003-024-06921-z)
Supplement: Supplementary file 4 — Description of Additional Supplementary Materials [file 42003_2024_6921_MOESM4_ESM.pdf]

Description of Additional Supplementary Files

File name: Supplementary Data 1

Description: Numerical Source Data and uncropped Western Blots
